# A Systematic Investigation into the Environmental Fate of Microcystins and The Potential Risk: Study in Lake Taihu

**DOI:** 10.3390/toxins8060170

**Published:** 2016-06-02

**Authors:** Junmei Jia, Qiuwen Chen, Torben L. Lauridsen

**Affiliations:** 1 Research Center for Eco-Environmental Sciences, Chinese Academy of Sciences, Beijing 100085, China; jiadao_mei@126.com; 2 Center for Eco-Environmental Research, Nanjing Hydraulic Research Institute, Nanjing 210029, China; 3 Department of Environmental Sciences, University of the Chinese Academy of Sciences, Beijing 100049, China; 4 Department of Bioscience, Aarhus University, Vejlsøvej 25, 8600 Silkeborg, Denmark; tll@bios.au.dk; 5 Sino-Danish Center for Education and Research (SDC), Beijing 100190, China

**Keywords:** microcystins, bioaccumulation, environment fate, health risk, Lake Taihu

## Abstract

A systematic investigation was conducted in Lake Taihu in autumn of 2013 and 2014, in order to understand the environmental fate of microcystins (MCs) and evaluate the health risk from MCs. Samples of water, algal cells, macrophytes, shrimps and fish were taken to detect MCs by HPLC-MS/MS after solid phase extraction. Widespread MC contamination in water, algal cells, macrophytes, shrimps and fish was found in Lake Taihu. The ubiquitous presence of MCs in water, algal cells and biota was found in 100% of samples. MC accumulation was in the order of primary producer > tertiary consumer > secondary consumer > primary consumer. The highest levels of MCs in macrophytes, shrimps and fish tissue were found in *Potamogeton* *maackianus*, *Exopalaemon* *modestus*, and *Hyporhamphus* *intermedius*, respectively. The MCs level in shrimps and the tissues of three fish species, *Neosalanx* *tangkahkeii* *taihuensis*, *Coilia* *ectenes* and silver carp, was closely linked to their dietary exposure. *Ceratophyllum* *demersum* L. was an ideal plant for introduction into lakes to protect against *Microcystis* blooms and MCs, due to its ability to absorb nutrients, accumulate large amounts of MCs and tolerate these toxins compared to other macrophytes. The average daily intakes (ADIs) of MCs for *Exopalaemon* *modestus* and three fish species, *Coilia* *ectenes*, *Hyporhamphus* *intermedius* and *Carassius* *carassius*, were all above the tolerable daily intakes (TDI) set by the World Health Organization (WHO), implying there existed potential threats to human health.

## 1. Introduction

Cyanobacteria, widely known as blue-green algae, are prokaryotes and are some of the earliest known organisms [1]. The presence of cyanobacteria dates back 3.5 billion years [1]. Cyanobacteria have a diverse group and are widely distributed throughout the world’s lakes and reservoirs [2]. Cyanobacterial blooms remain a growing worldwide concern [3,4,5]. The increase in cyanobacteria *Microcystis* producing hepatotoxic microcystins (MCs) has been highly worrisome [5]. Several genera of cyanobacteria including *Microcystis*, *Anabaena*, *Oscillatoria*, *Nostoc* and *Anabaenopsis* could produce MCs comprising a group of cyclic peptides [6]. Approximately 90 variants of MCs have been identified up to now [7].

MCs can have detrimental effects on phytoplankton, macrophytes, aquatic animals and even human beings. MCs inhibit the growth of aquatic organisms (including phytoplankton, macrophytes, invertebrates and vertebrates) [8,9], reduce fecundity [9] and accumulate into organisms, including mussels and fish. MCs have caused mass death of wild animals, livestock and aquatic life in many places in the world [10,11,12,13,14,15,16].

MCs have acutely toxic effects on humans such as jaundice, shock, and abdominal pain, and can cause death in human beings [1,17,18]. Chronic exposure to microcystins may cause different health effects, such as liver damage [19], promotion of liver tumour progression [20] and liver cancer [3,21,22]. MCs have been detected in the blood sera of Chinese fishermen with obvious liver damage, because of exposure to MCs via drinking water and aquatic products [23].

A series of field studies on MC contamination in water and aquatic animals were conducted in Lake Taihu around 2007 when a notorious heavy cyanobacterial bloom occurred [24,25,26]. During the algal bloom crisis in Lake Taihu in 2007, about 2 million people could not access tap water and had to rely on bottled water for 1 week [27,28]. In the years around 2007, Lake Taihu suffered from heavy blooms every summer. Extensive studies have been conducted on accumulation of MCs in fish, shrimps and snails [28,29,30,31,32,33], but systematic investigation of MCs in the food web including algal cells, macrophytes, shrimps and fish is scarce. Most of the studies were conducted during heavy cyanobacterial blooms, and all the results indicated that MC accumulation could pose high risks to higher trophic levels and human beings. But is it absolutely safe when there is no obvious bloom? A study in the James River Estuary indicated that potential health risks may exist in non-bloom periods [34]. Another study in the San Francisco Estuary indicated that *Microcystis*, even at low abundance, may have influences on fishery production via toxic and food web at multiple trophic levels [35]. Although there have not been large cyanobacterial blooms occurred in Lake Taihu since 2008, chlorophyll a concentration and average density of cyanobacteria in Lake Taihu have been increasing in recent years [36]. Thus, a systematic investigation the fate of MCs in a typical Chinese lake ecosystem is needed.

In the present study, six species of macrophytes, two species of shrimps and seven species of fish were investigated. The studied species are either dominant species or common species in Lake Taihu. The objective of this study was to: (1) explore the environmental fate of MCs in the food web associated with water, algal cells, macrophytes, shrimps and fish species during autumn in 2013 and 2014; (2) evaluate the risks that MCs pose to aquatic organisms and human beings.

## 2. Results

### 2.1. Spatial Differences of MCs in Water and Algal Cells of Lake Taihu

The average MC concentration in the water of Lake Taihu in autumn of 2013 was 130.4 ± 42.6 ng/L, and it was 140.2 ± 83.3 ng/L in autumn of 2014. The water in SC had the highest MC concentration of 202.0 ± 71.4 ng/L in autumn of 2013, while there was not much difference in MC levels in the water in the other three areas of Lake Taihu (Table 1). Water in MLB and SC had higher MC concentrations than in the other two areas in autumn of 2014 (Table 1). The ratios of MC-LR/MCs and MC-RR/MCs in water were similar in WC, LC and MLB in 2013, but the ratios of MC-LR/MCs in the water of SC was much higher than in water from WC, LC and MLB. The ratios of MC-LR/MCs and MC-RR/MCs in water were similar in WC, SC, LC and MLB in 2014.

The average MC concentrations in algal cells and total MCs were both higher in autumn of 2014 than in autumn of 2013 (*p* < 0.05). The average MC concentrations in algal cells in autumn of 2013 and 2014 were 629.3 ± 999.3 ng/L and 4104.5 ± 8086.7 ng/L, respectively. WC had higher MC level of 2491.0 ± 1486.1 ng/L in algal cells than SC and LC in 2013 (*p* < 0.05), and MLB had higher MC in cells, 18,063.3 ± 14,974.3 ng/L than SC and LC in 2014 (*p* < 0.05) (Table 1). The MC-RR/MCs ratio in algal cells was higher than the MC-LR/MCs and MC-YR/MCs ratios in algal cells of the four areas in 2013 and 2014, except for the MC-LR/MCs ratios in algal cells in LC and MLB in 2013.

### 2.2. Accumulation of MCs in Macrophytes of Four Areas of Lake Taihu

**Figure 1:**
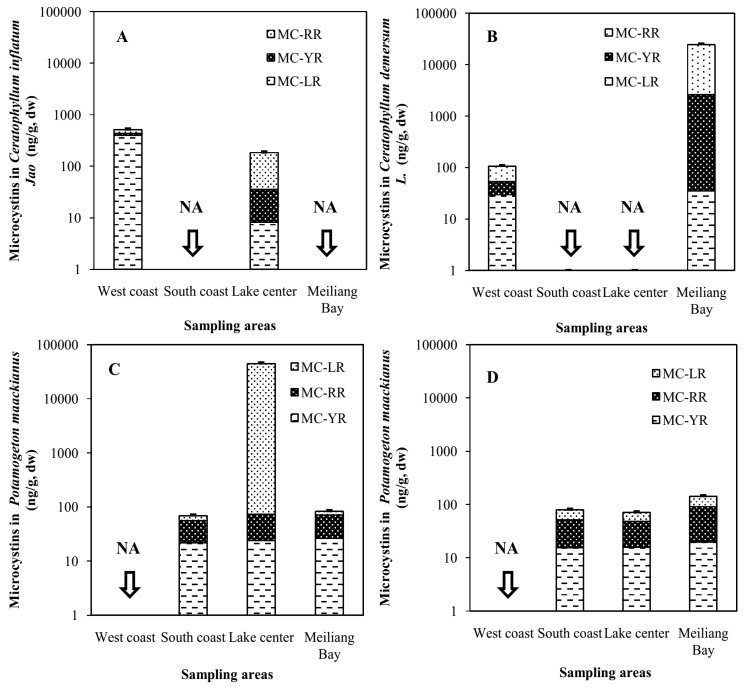
MC concentrations in three hydrophyte species from different areas of Lake Taihu in autumn of 2013 and 2014. (**A**) *Ceratophyllum inflatum Jao* in autumn of 2013; (**B**) *Ceratophyllum demersum* L. in autumn of 2013; (**C**) *Potamogeton maackianus* in autumn of 2013; (**D**) *Potamogeton maackianus* in autumn of 2014. NA, No data.

There were differences in MC accumulation among different macrophytes. The MC concentration in *Lemna minor* in LC in 2013 was as high as 8200.8 ± 282.1 ng/g dry weight (dw), while the MC content in *Ceratophyllum* *inflatum* *Jao* was 183.6 ± 6.3 ng/g dw. There were also differences among the same macrophytes sampled from different locations of Lake Taihu in 2013. The MC concentration of *Potamogeton* *maackianus* in LC in 2013 was 44,727.1 ± 1538.6 ng/g dw, while it was 68.4 ± 2.4 ng/g dw in SC of Lake Taihu. The MC concentration in *Ceratophyllum* *inflatum* *Jao* in WC was higher than in LC in 2013 (Figure 1A). The MC content in *Ceratophyllum* *demersum* L. in MLB was much higher than in WC (Figure 1B). In addition, the MC concentration in *Potamogeton* *maackianus* in LC of 2013 was much higher than in MLB and SC (Figure 1C). There were no great differences in MC contents in *Potamogeton* *maackianus* in MLB, LC and SC in 2014 (Figure 1D). Significant positive relationships were found between MCs in *Ceratophyllum* and MC-RR in algal cell (*p* < 0.05). Similar relation was also observed between MCs in *Ceratophyllum* and MCs in algal cell (*p* < 0.05) (Table 2). No significant relationships were found between MCs in *Potamogeton* *maackianus* and MCs in algal cells. Negative relationship was found between MCs in *Ceratophyllum* and total MCs in water, while positive relationship was found between MCs in *Potamogeton* *maackianus* and total MCs in water (Table 2).

### 2.3. Accumulation of MCs in Shrimp and Fish in MLB of Lake Taihu

**Figure 2:**
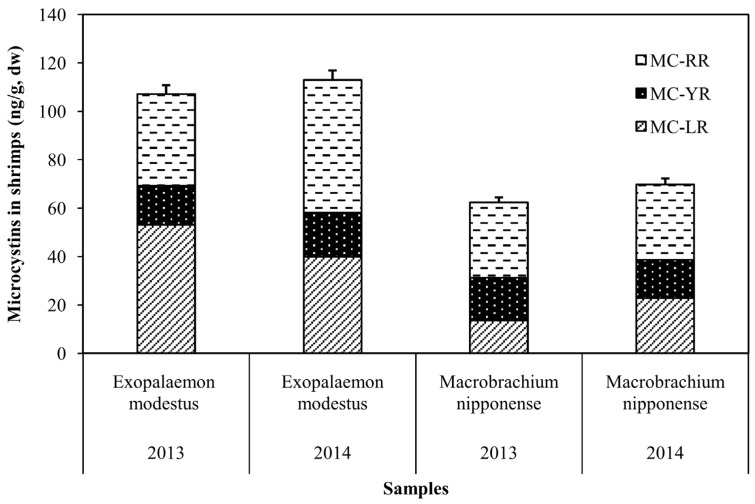
MC concentrations in two shrimp species sampled in MLB of Lake Taihu.

The mean MC concentration in shrimps of Lake Taihu in 2013 and 2014 was 84.7 ± 31.8 (mean ± SD) ng/g dw and 91.4 ± 30.5 ng/g dw, respectively. Both *Exopalaemon* *modestus* and *Macrobrachium* *nipponense* had slightly higher MC concentrations in 2014 than in 2013 (Figure 2). *Exopalaemon* *modestus* had higher MCs than *Macrobrachium* *nipponense* in both 2013 and 2014.

**Figure 3:**
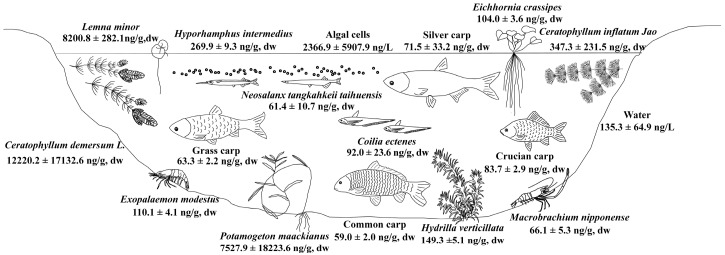
MC concentrations in the food web of Lake Taihu.

**Figure 4:**
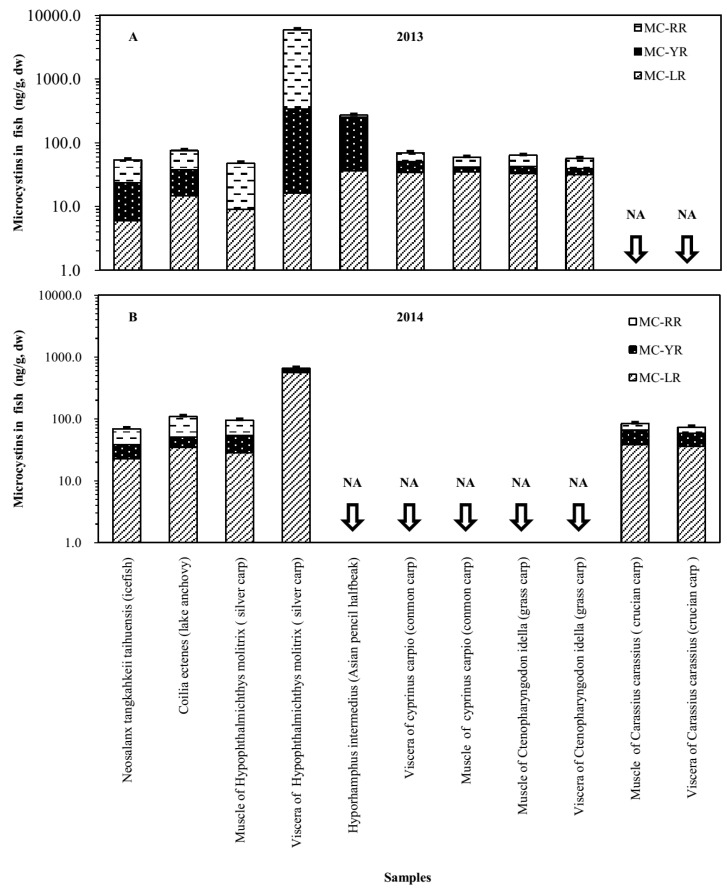
MC concentrations in fish sampled in MLB of Lake Taihu. NA, No data.

The average MC concentration in tissue of the same fish species in Lake Taihu sampled in 2013 and 2014 was 59.0 ± 14.4 ng/g dw and 90.9 ± 20.2 ng/g dw, respectively. The *Hyporhamphus* *intermedius* had MC levels of 269.9 ± 9.3 ng/g dw in its tissue, and *Coilia* *ectenes* had MC levels of 92.0 ± 23.6 ng/g dw (Figure 3). The *cyprinus* *carpio* had MC levels of 59.0 ± 2.0 ng/g dw in tissue. There were three fish species, *Neosalanx* *tangkahkeii* *taihuensis*, *Coilia* *ectenes* and silver carp, caught both in 2013 and 2014. The MC contents of the three species were all higher in 2014 than in 2013, except for those in viscera of silver carp (Figure 4).

### 2.4. Fate of MCs in the Food Web of Lake Taihu

**Figure 5:**
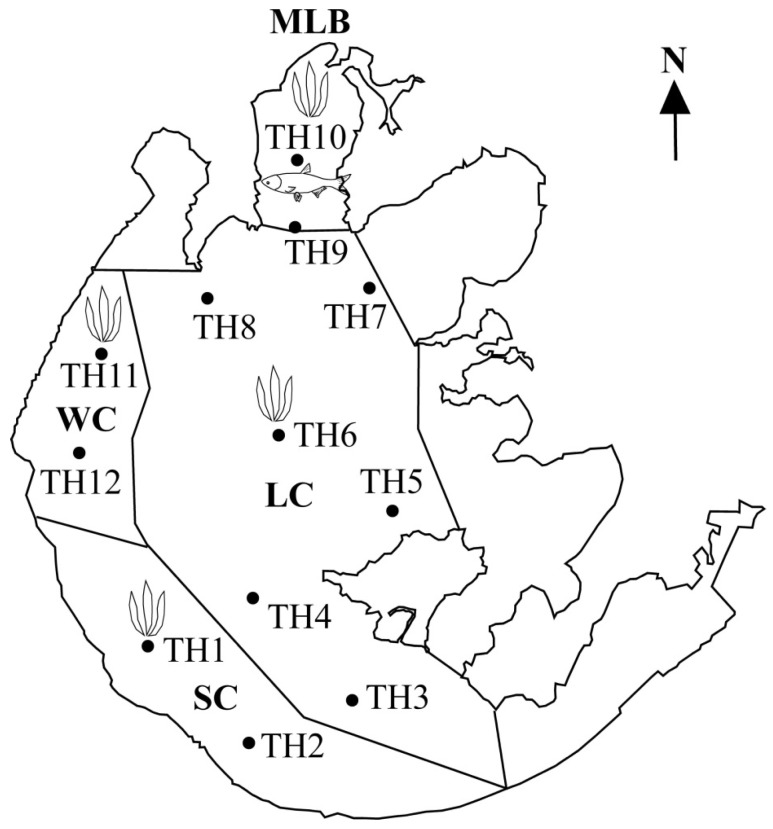
Sampling sites of water, hydrophytes and aquatic animals in Lake Taihu. TH, Taihu; MLB, Meiliang Bay; SC, South coast; WC, West coast; LC, lake centre.

The MC concentration in water of Lake Taihu ranged from 53.1 ng/L to 337.3 ng/L and MC in algal cells ranged from 118.2 ng/L to 28,651.7 ng/L. Aquatic organisms in this study all had MCs presence in their bodies (Figure 3). The MC contents in macrophytes of Lake Taihu ranged from 68.4 ± 2.4 ng/g dw to 44,727.1 ± 1538.6 ng/g dw. The MC contents in shrimp ranged from 62.3 ± 2.1 ng/g dw to 113.0 ± 3.9 ng/g dw, while MC in fish tissue ranged from 48.0 ± 1.7 ng/g dw to 269.9 ± 9.3 ng/g dw. Extremely high MC contents were found in macrophytes, such as *Ceratophyllum* *demersum* L., *Lemna minor* and *Potamogeton* *maackianus* (Figure 5). Relatively high MC contents were found in macrophytes, shrimps and fish, such as *Ceratophyllum* *inflatum* Jao, *Exopalaemon* *modestus* and *Hyporhamphus* *intermedius* (Figure 3). Relatively low MC contents were found in fish, such as common carp and grass carp (Figure 5). MC-RR was the main congener of MCs in algal cells, macrophytes, shrimps and fish. The MC-RR in algal cells, macrophytes, shrimps and fish of MLB accounted for 43% ± 25%, 65% ± 21%, 45% ± 8% and 50% ± 17% of MCs, respectively. The MC-LR in algal cells, macrophytes, shrimps and fish of MLB accounted for 46% ± 18%, 17% ± 18%, 35% ± 11% and 28% ± 20% of MCs, respectively. The MC-LR/MCs ratio of algal cells was significantly higher than the ratio of macrophytes in MLB (*p* < 0.05).

### 2.5. Risk of MCs to Local Residents

The exposure routes of MCs to local residents around Lake Taihu are mainly drinking water and food. A guideline value of 1.0 μg/L for MC-LR was set up in drinking water by [37]. If only the MC in water was taken into consideration, the MC concentration was much lower than 1.0 μg/L. However, the total MC in water, including the MC in cells, was much higher than 1.0 μg/L in WC, LC (2014) and MLB (2014). Dietary exposures of humans to MCs were calculated for 2 shrimp and 7 fish species (Table 3). The value of 0.04 μg/kg body mass per day derived from World Health Organization (WHO) [3] was used for tolerable daily intake (TDI) of MC-LR. According to medium lethal doses (LD_50_) in mice [38], MC-YR and MC-RR correspond to 0.4 and 0.2 MC-LR equivalents, respectively. The dry weights were converted to wet weight by coefficients of 5 [23]. Assuming that an average body mass of an adult is 60 kg in China and the daily consumption of fish muscle is 300g [24], the average daily intakes (ADIs) of MCs from eating *Exopalaemon* *modestus* and *Macrobrachium* *nipponense* would be 0.0625 and 0.031 μg MC-LR eq/kg body mass, respectively. The ADIs of *Exopalaemon* *modestus* and *Macrobrachium* *nipponense* are 1.6- and 0.8-fold the tolerable daily intake (TDI) proposed by the WHO. *Exopalaemon* *modestus* accumulated more MCs than *Macrobrachium* *nipponense*, but *Exopalaemon* *modestus* was more popularly served than *Macrobrachium* *nipponense* by local peoples. The mean daily intakes of MCs from eating *Neosalanx* *tangkahkeii* *taihuensis*, *Coilia* *ectenes*, *Hyporhamphus* *intermedius*, *Hypophthalmichthys* *molitrix*, *Carassius* *carassius*, *Cyprinus* *carpio* and *Ctenopharyngodon* *idella* would be 0.027, 0.042, 0.229, 0.032, 0.43, 0.021 and 0.025 μg MC-LReq/kg body mass, respectively, corresponding to 0.7-,1.1-, 5.7-, 0.8-, 1.1-, 0.5- and 0.6- times the TDI proposed by the WHO.

## 3. Discussion

Widespread MC contamination was observed among water, algal cells, macrophytes (6 species), shrimp (2 species) and fish (7 species) in Lake Taihu in our study. The ubiquitous presence of MCs in water (100% of samples), algal cells (100% of samples) and aquatic organisms (100% of samples) was surprising since there were no visible cyanobacterial blooms in most areas of Lake Taihu when we took those samples (The cyanobacteria abundance of Lake Taihu was 3.5 × 10^6^ cells/L and 7.5 × 10^7^ cells/L in autumn of 2013 and 2014, respectively). The average ratios of MC-LR/MCs in algal cells were higher than the ratios of shrimps, fish, and especially macrophytes (*p* < 0.05). These results indicated that organisms seem to be able to avoid toxic congeners or efficiently eliminate MCs in their bodies, given that MC-LR was the most toxic congener among MC-RR, MC-YR and MC-LR [38]. Extremely high MC contents were found in all macrophytes, which may result from a lack of efficient detox and elimination mechanisms in their tissue. The high concentration of MCs in macrophytes would pose a serious risk to the higher trophic level animals in the food web of Lake Taihu.

The MCs in phytoplankton may be eaten by primary consumers, such as filter fish, or secondary consumers via zooplankton [39]. The macrophytes would be directly eaten by herbivorous fish (grass carp) or ingested by other fish (crucian carp) and shrimp (*Macrobrachium* *nipponense*) as debris. There are some interesting results in the present study. The primary producers (phytoplankton and macrophyte) presented very high MC levels in their cells or tissues. The primary consumers (grass carp, silver carp, *etc.*) had lower MC concentrations in their tissues. Then the secondary consumers (*Exopalaemon* *modestus*, *Macrobrachium* *nipponense*, icefish) had higher MCs in their tissues than the primary consumers. The highest level consumers in this study (Asian pencil halfbeak, lake anchovy, *etc.*) had higher MC concentrations in tissue than the secondary consumers. This might be because the primary consumers, for example silver carp, ingest algal cells directly, and they may have evolutionary adaptations to possess high resistance to MC exposure and mechanisms to eliminate MCs in their body to counteract MCs [40]. The grass carp prefer to eat *Hydrilla* *verticillata* rather than *Ceratophyllum* *demersum* [41] and the former species accumulated much lower levels of MCs in this study. The higher level consumers, such as *Hyporhamphus* *intermedius* and *Coilia* *ectenes*, do not have a mechanism to counteract MCs, but they have an inevitable intake of MCs through predation, and thus they present high MC levels in the body.

In the present study, considerable differences were found among macrophytes sampled from different areas of Lake Taihu, and all these differences arose from a distinct increase in the concentration of either MC-RR or MC-LR (Figure 1). There were great differences in MCs among different macrophyte species, which was also found by previous researchers [25,42]. The two floating macrophytes *Ceratophyllum* *demersum* L. and *Lemna minor* had the highest MC concentrations in their tissues. This is because they float on the surface of water together with algal cells or scum, and they can absorb the MCs rapidly as soon as MCs were released into water from algal cells [43,44]. This was further demonstrated by the significant positive relationship between MCs in *Ceratophyllum* and MCs in algal cells. Besides, these two plants have the ability to absorb large amounts of nutrients [45,46] and the MCs may enter into the plant by a similar pathway as nutrients [47]. These two plants should have priority when macrophytes are introduced to lakes to protect against *Microcystis* blooms and MCs, because they play an vital role in elimination of MCs via accumulation in plant tissues, and *Ceratophyllum* *demersum* L. has higher tolerance to MCs compared to other macrophytes such as *Hydrilla* *verticillata* [48]. The negative relationship between MCs in *Ceratophyllum* and total MCs in water also indicated that the presence of *Ceratophyllum* *demersum* L. contributed to the lower total MCs in water columns. Another macrophyte, *Potamogeton* *maackianus*, one of dominant macrophytes in Lake Taihu, also showed high concentrations of MCs, indicating that they may play a vital role in elimination of MCs in Lake Taihu.

Results of both shrimps species showed that living in an environment with higher levels of MCs (the MC levels in water and algal cells were higher than in other samples) could lead to higher MC levels accumulated in the bodies of shrimp; a similar result was found in the James River Estuary for fish and shellfish [34]. The results suggested that levels of MC in tissues of shrimp, shellfish and some fish species could be associated with dietary exposure for consumers. The results for the three fish species (*Neosalanx* *tangkahkeii* *taihuensis, Coilia* *ectenes* and silver carp) caught in both 2013 and 2014 showed that fish living in an environment with higher MC concentrations had more MCs in their muscle, which was similar to the results for shrimp. The dominant species *Hyporhamphus* *intermedius* and *Coilia* *ectenes*, which are both pelagic species, accumulated higher MC levels in their body than other fish species. This may be because they live in the upper layer of the water body and thus have more access to algal cells. Meanwhile, they do not have a detox ability similar to that of silver carp. Therefore, they may pose potential risks to higher trophic animals and human beings [49,50].

Both laboratory and field studies indicate that the presence of MCs in aquatic ecosystemshad adverse impacts on aquatic plants [42,51,52,53,54]. Exposure to MCs could decrease the growth rate of *Lemna gibba* [51], *Lemna minor* and *Wolffia arrhiza* [54]. Increase of MCs in the water can cause oxidative stress and had adverse impacts on metabolism of aquatic plants by suppressing the soluble protein contents [53]. MCs had sublethal effects on aquatic fauna, including zooplankton [55,56], macroinvertebrates and aquatic insects [57,58,59,60]. Malbrouck and Kestemont reviewed the effect of MCs on fish and discussed the potential consequences in aquatic ecosystems [61]. In early life stages of fish, exposure to MCs had adverse effects on embryonic hatching, decreased survival and growth rate, and resulted in histopathological effects. In adults and juveniles, MC exposure could affect growth rate and had histopathological effects on several organs of fish [61,62,63]. Besides, the carnivorous fish are more sensitive to MCs than phytoplanktivorous fish [24,63]. Christoffersen reviewed the values of microcystin concentrations that have proved lethal or caused significant changes in physiological or behavioural processes [64]. Fish are the most tolerant (0.5–20 µg toxin·mL^−1^), phytoplankton are intermediately tolerant (0.05–1 µg toxin mL^−1^), and macrophytes (*Lemna* and *Elodea*) and nano-sized protozoa (heterotrophic nanoflagellates) are the least tolerant organisms (0.001–0.05 µg toxin·mL^−1^). According to the MC levels in this study, MCs could cause significant effects on physiological or behavioural processes, or even death to some macrophytes and nano-sized protozoa in Lake Taihu. Other studies in Lake Taihu also showed that the *Microcystis* blooms strongly inhibited the population growth of the water flea, *Daphnia magna*, by reducing their survival rates, individual growth rate, or gross fecundity [65]. In the worst case, cyanobacterial blooms can eliminate filter-feeding zooplankton and change the competitive relations among zooplankton, which may undermine the food chain and destroy the ecological balance in aquatic ecosystems [65,66].

Although the MC concentration in the water of Lake Taihu was much lower than the WHO guideline value, the MC concentration in algal cells was several times of the guideline value which maybe released into water during drinking water treatment processes [67,68] and pose risks to human health. From Table 3, the ADIs of MCs for one shrimp and three fish species exceeded the provisional TDI proposed by WHO, especially *Hyporhamphus* *intermedius*. Compared to previous studies sampled in blooming periods, the mean daily intakes of MCs were estimated to be 2.2 times the recommended TDI for MC-LR in Lake Taihu [69]. The MCs consumption risk of fish muscle in blooming periods was much higher than in non-blooming periods. However, some fish such as *Coilia* *ectenes* and *Hyporhamphus* *intermedius* collected from Lake Taihu during non-blooming periods could also pose risks to human beings. However, there are few studies considering the consumption risk of MC for children. Mulvenna *et al.* assessed the consumption risk of MCs taking adults and children separately [70]. The health guideline values in seafood for MC derived from the study were lower for children than for adults, indicating that safe seafood for adults is not necessarily safe for children.

## 4. Conclusions

Widespread MC contamination in water, algal cells, macrophytes, shrimps and fish was observed in Lake Taihu. MC accumulation followed the order of primary producer > tertiary consumer > secondary consumer > primary consumer. The highest MCs in macrophytes, shrimp and fish tissue were found in *Potamogeton* *maackianus*, *Exopalaemon* *modestus*, and *Hyporhamphus* *intermedius*, respectively. MCs in their tissues can be associated with the dietary exposure of shrimp and three fish species: *Neosalanx* *tangkahkeii* *taihuensis*, *Coilia* *ectene*s and silver carp. Further studies are necessary to identify the relationship between dietary exposure and MC accumulation in tissue. *Ceratophyllum* *demersum* L. is an ideal plant to protect against *Microcystis* blooms and MCs in lakes due to its ability to absorb nutrients, accumulate large amounts of MCs and exhibittolerance to MCs, compared to other macrophytes. The ADIs of MCs for *Exopalaemon* *modestus* and three fish species, *Coilia* *ectenes*, *Hyporhamphus* *intermedius* and *Carassius* *carassius*, were all above the TDI proposed by WHO, which may pose threats to the health of local residents. Routine monitoring should be applied in Lake Taihu to avoid consumption of contaminated water, shrimps and fish.

## 5. Materials and Methods

### 5.1. Study Area and Sampling Sites

The most polluted part of Lake Taihu was selected as the study area according to Lake Taihu’s eutrophication status [33]. Water and phytoplankton were both sampled at 12 sites (Figure 5). Macrophytes were sampled in the West coast (WC), South coast (SC), lake centre (LC) and Meiliang Bay (MLB) (Figure 5). Shrimps and fish were sampled in MLB (Figure 5).

### 5.2. Sampling and Processing

Water samples of Lake Taihu were taken on 24 September 2013 and 24 September 2014, when cyanobacterial blooms frequently appeared in the years before. Surface water samples of 2 L were collected at 12 sites using plastic bottles and were stored at 4 °C in the dark before laboratory analysis (Figure 5).

In the laboratory, one litre of the sample water was pre-filtered using glass fibre filters (GF/C, Whatman, GE Healthcare UK Limited, Little Chalfont, Bucks, UK) under vacuum within 1 day. The filtered water was extracted by solid phase extraction (SPE) according to the procedures from Dai *et al*. [71]. The glass fibre filters were used to detect MCs in the algal cells. The extraction of MCs in algal cells followed a previous method [72].

Floating and rooted macrophytes were collectedon 24 September 2013 and 24 September 2014. Six macrophytes were sampled: *Ceratophyllum* *inflatum* Jao, *Ceratophyllum* *demersum* L., *Hydrillaverticillata*, *Lemna minor*, *Potamogeton* *maackianus* and *Eichhornia* *crassipes* (Figure 5). At least 3 individuals per species were collected. Two species of shrimp, *Exopalaemon* *modestus* and *Macrobrachium* *nipponense*, were sampled with the help of local fisherman on 24 September 2013 and 24 September 2014. Seven fish species including *Hyporhamphus* *intermedius* (Asian pencil halfbeak), *Neosalanx* *tangkahkeii* *taihuensis* (icefish), *cyprinus* *carpio* (common carp), *Coilia* *ectenes* (lake anchovy), *Ctenopharyngodon* *idella* (grass carp), *Carassius* *carassius* (crucian carp) and *Hypophthalmichthys* *molitrix* (silver carp) were also sampled with help from local fisherman on 24 September 2013 and 24 September 2014 (Figure 5). The weight and length of each shrimp and fish was measured. Then fish was immediately dissected. At least 3individuals per species were prepared. The individuals were mixed and frozen at −20 °C for storage before MCs quantification. For the fish samples of *Ctenopharyngodon* *idella* (grass carp), *cyprinus* *carpio* (common carp), *Carassius* *carassius* (crucian carp), and *Hypophthalmichthys* *molitrix* (silver carp) muscle and visceral (the whole inside part of the fish) tissues were taken for analysis. For other species, the whole fish was taken.

Samples of tissues were lyophilized, crushed and homogenized. Approximately 0.4 g dry weight (dw) portions were extracted three times with 10 mL water:butanol:methanol=1:4:15 while stirring for 24 h. The extract procedures followed the method from Xie *et al*. [73].

### 5.3. MCs Quantification

Microcystins were quantified using the method of [74] with some modification [33]. Samples in MeOH were injected into an Agilent 6460 QQQ HPLC-MS/MS equipped with an Agilent Zorbax Eclipse Plus column (C18, 2.1 mm × 50 mm, 1.8 μm). Separation was accomplished using two phases. Phase A was MeOH and phase B was water with 0.1% formic acid (FA). The flow rate of the mobile phase was 0.5 mL/min. The temperature of column oven was set at 40 °C. For more detailed information please see [33]. Instrument control, data processing and data analysis were conducted using Agilent MassHunter software B.04.00 (Agilent Technologies, Inc., Santa Clara, CA, USA, 2011). The MC concentrations of the samples from Lake Taihu were quantified by comparing the peak areas of the test samples to those of MC-LR, MC-YR and MC-RR standards from AXXORA Europe, Switzerland.

### 5.4. Statistical Analysis

Data were processed using SPSS 17.0 (SPSS, Inc., Chicago, IL, USA, 2008). Analysis of variance (ANOVA) was applied to find whether significant variation of MCs exists in water, algal cells and their total MCs between WC, SC, LC and MLB. ANOVA was also applied to find whether significant variation of MCs exists in water, algal cells and the total MCs between different years. Pearson correlation analysis was used to examine whether there were significant relationships between MC in macrophytes and MC in water and algal cells. Data were transformed by log(*X* + 1) when the ANOVA and correlation analysis were conducted. The criterion for significance was *p* < 0.05 and *p* < 0.01.

## Figures and Tables

**Table 1:** MC concentrations in water and algal cells in different areas of Lake Taihu (*p* < 0.05).

Year	Areas	MC-RR (ng/L)	MC-LR (ng/L)	MC-YR (ng/L)	MCs (ng/L)	MC-RR Cell (ng/L)	MC-LR Cell (ng/L)	MC-YR Cell (ng/L)	MCs Cell (ng/L)	Total MCs (ng/L)
2013	WC	46.9 ± 17.2	44.9 ± 17.7	36.9 ± 10.9	128.8 ± 24.0	1796.2 ± 2300.6	248.4 ± 266.5	446.4 ± 548.0	2491.0 ± 1486.1 ^b^	2619.8 ± 1510.1 ^b^
2013	SC	37.5 ± 0.31	113.9 ± 66.9	50.6 ± 4.8	202.0 ± 71.4	87.0 ± 62.2	20.9 ± 0.1	39.0 ± 23.8	146.9 ± 38.6 ^a^	348.9 ± 110.0 ^ab^
2013	LC	35.5 ± 0.8	47.0 ± 17.0	33.3 ± 5.6	115.8 ± 16.2	60.5 ± 46.0	115.2 ± 111.3	27.8 ± 3.9	203.6 ± 113.2 ^a^	319.3 ± 108.3 ^a^
2013	MLB	36.3 ± 5.1	38.2 ± 2.7	30.0 ± 0.5	104.5 ± 7.3	137.6 ± 148.7	345.4 ± 334.8	44.1 ± 30.6	527.2 ± 452.9 ^ab^	631.7 ± 445.6 ^ab^
2014	WC	35.1 ± 2.5	27.8 ± 3.3	14.4 ± 0.5	77.3 ± 5.3	626.1 ± 365.9	280.9 ± 165.5	43.7 ± 18.7	950.8 ± 550.1 ^AB^	1028.1 ± 555.4 ^A^
2014	SC	90.7 ± 15.4	95.1 ± 42.2	20.7 ± 0.7	206.4 ± 58.3	487.5 ± 481.7	261.2 ± 235.6	35.0 ± 24.8	783.7 ± 742.1 ^A^	990.1 ± 800.4 ^A^
2014	LC	53.8 ± 24.1	45.3 ± 20.0	13.9 ± 7.0	112.9 ± 47.6	1087.7 ± 1552.1	454.0 ± 620.4	68.2 ± 76.5	1609.8 ± 2248.5 ^A^	1722.8 ± 2291.0 ^A^
2014	MLB	111.5 ± 94.0	86.5 ± 66.0	20.5 ± 8.0	218.5 ± 168.0	11,624.8 ± 9689.1	5679.4 ± 4597.4	759.1 ± 687.7	18,063.3 ± 14,974.3 ^B^	18,281.9 ± 15,142.3 ^B^

^a, ab, b^ are symbols whether there were significant differences in different areas of Lake Taihu in 2013; A, AB and B are symbols whether there were significant differences in different areas of Lake Taihu in 2014. The MCs in the area marked with “a” had significant differences with the area marked with “b”, while it had no significant differences with the area marked with “ab”. This applies to “A”, “B” and “AB” as well.

**Table 2:** Pearson correlations between MC in macrophytes and MC in water and algal cells.

Correlations	*Ceratophyllum*				*Potamogeton maackianus*			
	MC-RR	MC-LR	MC-YR	MCs	MC-RR	MC-LR	MC-YR	MCs
MC-RR (water)	0.83	0.06	−0.36	−0.51	0.55	−0.53	−0.58	−0.38
MC-LR (water)	−0.19	0.36	−0.92	0.64	−0.02	−0.24	−0.33	−0.37
MC-YR (water)	0.54	0.21	−0.69	−0.09	−0.19	−0.01	0.59	0.19
MCs(water)	0.1	−0.35	0.92	−0.57	0.27	−0.46	−0.4	−0.4
MC-RR (cells)	−0.93	0.21	−0.33	0.96 *	−0.27	0.02	0.36	−0.45
MC-LR (cells)	0.95	−0.18	0.24	−0.93	−0.03	0.03	0.52	0.5
MC-YR (cells)	0.95 *	−0.17	0.22	−0.92	−0.14	0.35	0.28	0.71
MCs(cells)	−0.76	0.32	−0.65	0.98 *	−0.29	−0.07	0.44	−0.54
Total MC-RR	−0.36	−0.27	0.8	−0.12	−0.56	0.51	−0.07	0.21
Total MC-LR	−0.64	−0.16	0.6	0.24	−0.68	0.49	−0.38	−0.12
Total MC-YR	−0.89	0.003	0.22	0.65	−0.07	−0.31	−0.11	−0.55
Total MCs	0.95	−0.09	−0.01	−0.8	−0.08	0.39	0.04	0.75

* *p* < 0.05.

**Table 3:** Consumption risk assessment using tolerable daily intake (TDI).

Species	MC in Edible Parts (MC-LReq ng/g)	Daily Intake for a 60 kg Person (MC-LR eqμg/kg Body Weight)	×Lifetime TDI (0.04 μg/kg Body Weight/day)	Max Daily Intake Not Exceeding the Guideline (g)
*Exopalaemon modestus*	12.5	0.0625	1.6	192
*Macrobrachium nipponense*	6.2	0.031	0.8	387
*Neosalanx tangkahkeii taihuensis*	5.4	0.027	0.7	444
*Coiliaectenes*	8.4	0.042	1.1	286
*Hyporhamphusintermedius*	45.8	0.229	5.7	52
*Hypophthalmichthys molitrix*	6.3	0.032	0.8	381
*Carassius carassius*	8.6	0.043	1.1	279
*Cyprinus carpio*	4.2	0.021	0.5	571
*Ctenopharyngodon idella*	4.9	0.025	0.6	490
